# Efficacy of anakinra on rebound of multisystem inflammatory syndrome

**DOI:** 10.1111/ped.15337

**Published:** 2022-11-04

**Authors:** Giacomo Brisca, Camilla Olcese, Maria Elena Derchi, Gianluca Trocchio, Roberta Caorsi, Andrea Moscatelli, Marco Gattorno

**Affiliations:** ^1^ Terapia Semintensiva IRCCS Istituto Giannina Gaslini Genoa Italy; ^2^ Dipartimento di Neuroscienze, Riabilitazione, Oftalmologia, Genetica e Scienze Materno‐Infantili (DiNOGMI) Università degli Studi di Genova Genoa Italy; ^3^ U.O.C. Cardiologia IRCCS Istituto Giannina Gaslini Genoa Italy; ^4^ UOC Clinica Pediatrica e Reumatologia IRCCS Istituto Giannina Gaslini Genoa Italy; ^5^ UOSD Centro Malattie Autoinfiammatorie e Immunodeficienze IRCCS Istituto Giannina Gaslini Genoa Italy

**Keywords:** acute pericarditis, anakinra, MIS‐C, multisystem inflammatory syndrome in children, SARS‐CoV‐2

Multisystem inflammatory syndrome in children (MIS‐C) is a rare, potentially life‐threatening inflammatory condition that can develop in children 4–6 weeks after infection with severe acute respiratory syndrome coronavirus 2 (SARS‐CoV‐2).

It is known that early anti‐inflammatory therapy can be lifesaving but the optimal therapeutic regimen for MIS‐C has not yet been established.

Most patients have been treated with intravenous immunoglobulin (IVIG) alone or in combination with steroids, and biologic drugs (infliximab, anakinra) were given in severe or refractory cases.^1^

Data on short‐term outcomes are reassuring;^2^ however long‐term cardiac and non‐cardiac sequelae are currently poorly understood. In particular, information on the possibility of recurrence is scarce and limited to case reports.^3^

Here, we report on an unvaccinated and previously healthy 11‐year‐old boy who initially presented to our emergency department with a 2 day history of fever, vomiting, and chest and abdominal pain. Blood examination revealed neutrophilic leukocytosis (16 490/mm^3^), lymphopenia (790/mm^3^), and increased C‐reactive protein (CRP) levels (14 mg/dL, normal values <0,46). Abdominal ultrasonography showed peritoneal fluid and mild enlargement of the appendix, which had a folded appearance and an internal appendicolith. Laparoscopic appendicectomy was performed with evidence of mild appendicitis but persistence of high fever, and chest and abdominal pain with new onset of macular rash on the trunk were observed. Blood tests showed a further increase of acute phase reactants (Fig.1), with concomitant rise of ferritin (713 ng/mL, normal values 20–200) and N‐terminal fragment brain natriuretic peptide (2,100 pg/mL, normal values 0–125).

**Fig. 1:**
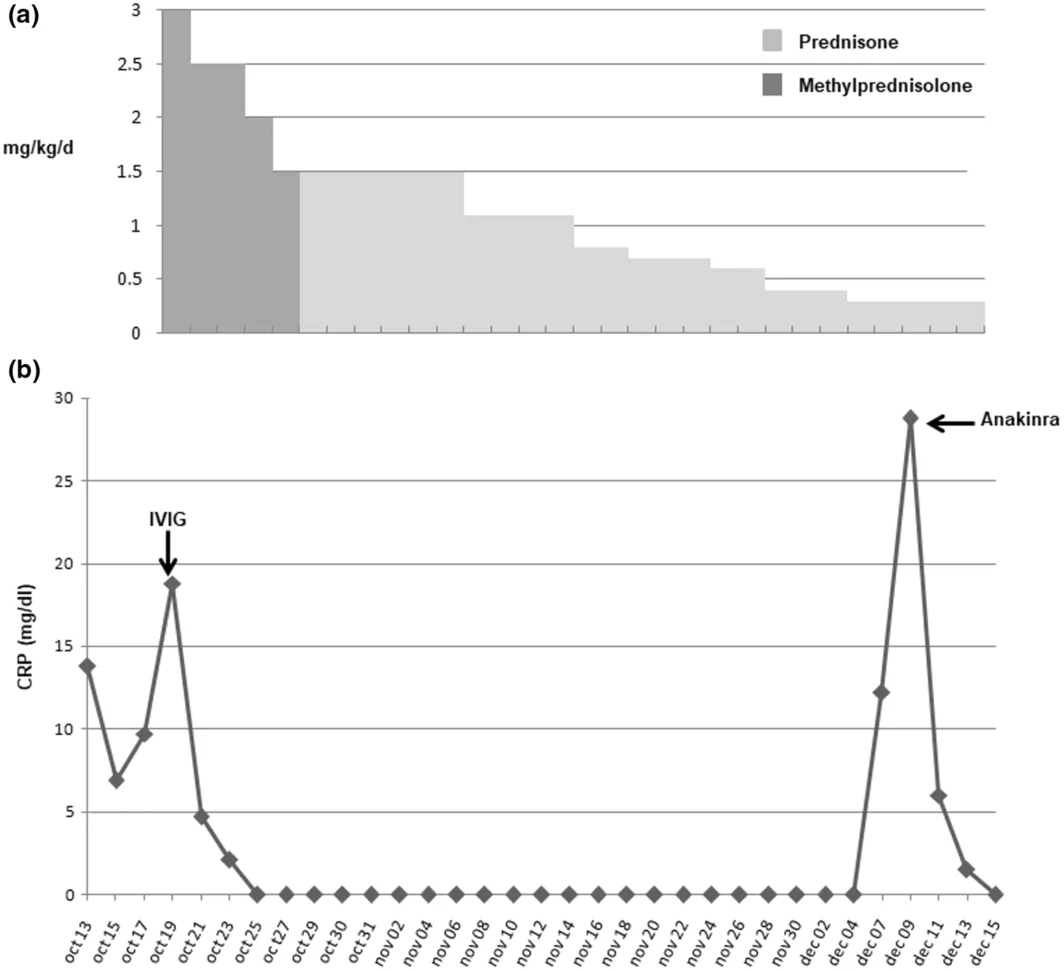
Disease flares, C‐reactive protein levels (CRP), therapeutic interventions with IV immunoglobulin, and Anakinra (panel a), and steroids (panel b) during the disease course. (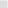), prednisone; (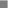), methylprednisolone.

A chest radiograph showed right pleural effusion. A new abdominal ultrasonography was negative. Echocardiography detected left coronary dilatation (Z score + 2.8) and pericardial thickening and effusion with preserved left ventricular function.

A polymerase chain reaction for SARS‐CoV‐2 on a nasopharyngeal swab was negative but immunoglobulin G (IgG) (6 AU/mL, normal values <1) and immunoglobulin M (IgM) (4,796 AU/mL, normal values <1) SARS‐CoV‐2 were positive, supporting a diagnosis of MIS‐C. Neither the child nor the family members reported symptoms consistent with COVID‐19 in the weeks before. However, numerous cases had been described among schoolmates.

According to our institutional treatment protocol^4^ and to standard clinical practice guidelines of the American College of Rheumatology,^1^ IVIG and IV methylprednisolone (3 mg/kg/day) were started with prompt clinical improvement and CRP normalization after 6 days (Fig.1).

On day 20 the child was discharged with tapering steroid treatment.

One month later, while he was assuming 0.2 mg/kg/day of oral prednisone, the child was readmitted for a relapse of fever and chest pain with echocardiographic evidence of acute pericarditis and laboratory evidence of acute inflammation (Fig.1).

After informed consent, intravenous anakinra (3 mg/kg/day) in association with an unchanged steroid dosage was initiated, leading to a dramatic clinical response with a fast and persistent normalization of the laboratory parameters (Fig.1).

At follow up, 6 months later, the child was in good general condition on anakinra (3 mg/kg/day) and prednisone (0.07 mg/kg/day) therapy with negative echocardiographic findings. Acute phase reactants were in the normal range.

Anakinra is known to be an effective therapeutic agent in MIS‐C,^1^, ^4^ especially in children who do not respond to IVIG and corticosteroids. The paediatric inflammatory multisystem syndrome temporally associated with SARS‐CoV‐2 arm of the randomised evaluation of COVID‐19 therapy trial is currently evaluating its efficacy for refractory disease.^5^

Although currently recommended as third‐line therapy (1), it is likely that in our case a more aggressive initial treatment approach with an early use of an interleukin‐1 (IL‐1) inhibitor, would have avoided disease rebound.

Our group has already shown that a timely use of anakinra as first‐line therapy in more severe cases may be decisive in stopping the hyperinflammation that underlies the disease and preventing the need for escalation of care.^4^

Although limited to a single case description, our report may further suggest that the finding of acute pericarditis should be considered as a risk factor for disease rebound of MIS‐C, suggesting in this case a more aggressive initial anti‐inflammatory therapeutic approach.

Finally, our report adds evidence for the efficacy of an IL‐1 inhibitor not only in children with life‐threatening presentations or who do not respond to first‐line treatment, but even in those who may develop a rebound or recurrence of MIS‐C.

We underline the need for a better definition of therapeutic strategies and a univocal allocation of anakinra within the therapeutic algorithm.

Follow‐up studies combined with the results of the ongoing trial will help clarify long‐term prognosis and recurrence probabilities.

This study was approved by the Regione Liguria Ethical Board (IRB# 370/2020).

## Disclosure

The authors declare no conflict of interest.

## Author contributions

G.B., M.D., R.C., and G.T. contributed to the conception and design of this study. C.O. collected, analyzed data and drafted the figure. G.B. drafted the manuscript. A.M. and M.G. critically reviewed the manuscript and supervised the whole study process. All authors read and approved the final manuscript.
